# Essential Oils’ Chemical Characterization and Investigation of Some Biological Activities: A Critical Review

**DOI:** 10.3390/medicines3040025

**Published:** 2016-09-22

**Authors:** Wissal Dhifi, Sana Bellili, Sabrine Jazi, Nada Bahloul, Wissem Mnif

**Affiliations:** 1 UR Ecophysiologie Environnementale et Procédés Agroalimentaires, Institut Supérieur de Biotechnologie de Sidi Thabet, BiotechPole de Sidi Thabet, Université de la Manouba, Ariana 2020, Tunisia; wissal_d2002@yahoo.fr; 2 LR11-ES31 Laboratory of Biotechnology and Valorisation of Bio-GeoRessources (BVBGR), Higher Institute of Biotechnology of Sidi Thabet (ISBST), Biotechpole Sidi Thabet, University of Manouba, Ariana 2020, Tunisia; bel-sana@hotmail.fr (S.B.); jazi.gb@gmail.com (S.J.); nada.bahloul@gmail.com (N.B.); 3 Faculté des Sciences de Bizerte, Jarzouna-Bizerte, Université de Carthage, Carthage 7021, Tunisia; 4 Faculty of Sciences and Arts in Balgarn, PO BOX 60 Balgarn, University of Bisha, Sabt Al Alaya 61985, Saudi Arabia

**Keywords:** essential oils, chemical composition, biological activities

## Abstract

This review covers literature data summarizing, on one hand, the chemistry of essential oils and, on the other hand, their most important activities. Essential oils, which are complex mixtures of volatile compounds particularly abundant in aromatic plants, are mainly composed of terpenes biogenerated by the mevalonate pathway. These volatile molecules include monoterpenes (hydrocarbon and oxygenated monoterpens), and also sesquiterpenes (hydrocarbon and oxygenated sesquiterpens). Furthermore, they contain phenolic compounds, which are derived via the shikimate pathway. Thanks to their chemical composition, essential oils possess numerous biological activities (antioxidant, anti-inflammatory, antimicrobial, etc…) of great interest in food and cosmetic industries, as well as in the human health field.

## 1. Introduction

The attraction of medicinal and aromatic plants is continuously growing due to increasing consumers demand and interest in these plants for culinary, medicinal, and other anthropogenic applications.

As consumers are becoming more and more informed about issues of food, health, and nutrition, they are also becoming aware of the benefits and potential applications of medicinal and aromatic plants and their metabolites. These plants produce a large variety of secondary metabolites; among them, essential oils.

Despite their rich and complex composition, the use of essential oils remains wide and limited to the cosmetics and perfumery domains. It is worthy to develop a better understanding of their chemistry and the biological properties of these extracts and their individual components for new and valuable applications in human health, agriculture, and the environment. Essential oils could be exploited as effective alternatives or complements to synthetic compounds of the chemical industry, without inducing the same secondary effects.

## 2. Definition of Essential Oils

The term essential oil dates back to the sixteenth century and derives from the drug *Quinta essentia*, named by Paracelsus von Hohenheim of Switzerland [1]. Essential oils or “essences” owe their name to their flammability. Numerous authors have attempted to provide a definition of essential oils. The French Agency for Normalization: Agence Française de Normalisation (AFNOR) gives the following definition (NF T 75-006): “The essential oil is the product obtained from a vegetable raw material, either by steam distillation or by mechanical processes from the epicarp of Citrus, or “dry”” distillation. The essential oil is then separated from the aqueous phase by physical means [2]. This definition encompasses products obtained always from vegetable raw material, but using other extraction methods, such as using non-aqueous solvents or cold absorption. Thus, we can define four types of products [3].

Essential oils are soluble in alcohol, ether, and fixed oils, but insoluble in water. These volatile oils are generally liquid and colorless at room temperature. They have a characteristic odor, are usually liquid at room temperature and have a density less than unity, with the exception of a few cases (cinnamon, sassafras, and vetiver). They have a refractive index and a very high optical activity. These volatile oils contained in herbs are responsible for different scents that plants emit. They are widely used in the cosmetics industry, perfumery, and also aromatherapy. The latter is intended as a therapeutic technique including massage, inhalations, or baths using these volatile oils. The last key will serve as chemical signals allowing the plant to control or regulate its environment (ecological role): attraction of pollinating insects, repellent to predators, inhibition of seed germination, or communication between plants (emission signals chemically signaling the presence of herbivores, for example). Moreover, EOs also possesses antifungal or insecticide and deterrent activities. All parts of aromatic plants may contain essential oils as follows:

- Flowers, of course, including: orange, pink, lavender, and the (clove) flower bud or (ylang-ylang) bracts,
- Leaves, most often, including: eucalyptus, mint, thyme, bay leaf, savory, sage, pine needles, and tree underground organs, e.g., roots (vetiver),
- Rhizomes (ginger, sweet flag),
- Seeds (carvi, coriander),
- Fruits, including: fennel, anise, Citrus epicarps,
- Wood and bark, including: cinnamon, sandalwood, rosewood.

## 3. Chemistry of Essential Oils

Essential oils are produced by various differentiated structures, especially the number and characteristics of which are highly variable. Essential oils are localized in the cytoplasm of certain plant cell secretions, which lies in one or more organs of the plant; namely, the secretory hairs or trichomes, epidermal cells, internal secretory cells, and the secretory pockets. These oils are complex mixtures that may contain over 300 different compounds [4]. They consist of organic volatile compounds, generally of low molecular weight below 300. Their vapor pressure at atmospheric pressure and at room temperature is sufficiently high so that they are found partly in the vapor state [5,6]. These volatile compounds belong to various chemical classes: alcohols, ethers or oxides, aldehydes, ketones, esters, amines, amides, phenols, heterocycles, and mainly the terpenes. Alcohols, aldehydes, and ketones offer a wide variety of aromatic notes, such as fruity ((E)-nerolidol), floral (Linalool), citrus (Limonene), herbal (γ-selinene), etc.

**Figure 1:**
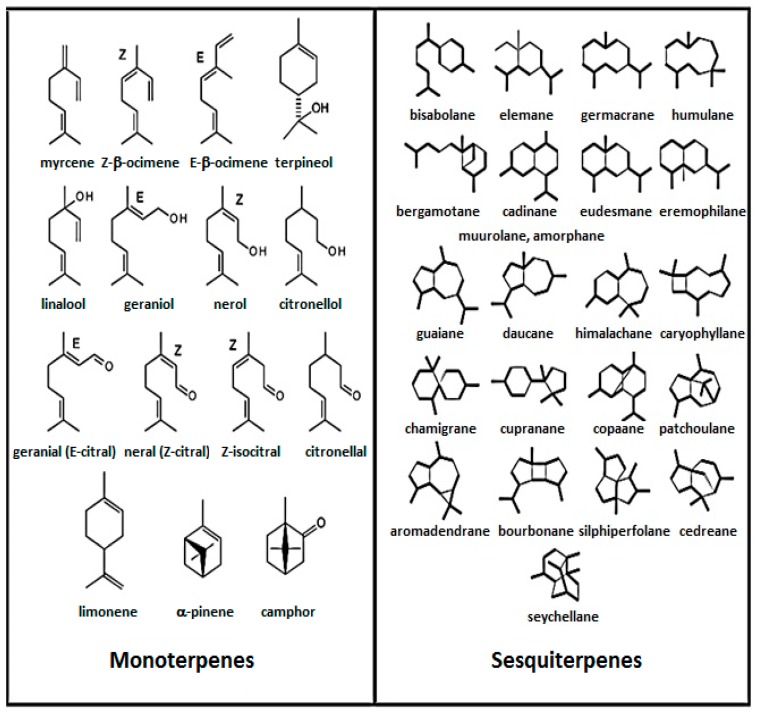
Structures of some terpenes.

Furthermore, essential oil components belong mainly to the vast majority of the terpene family (Figure 1). Many thousands of compounds belonging to the family of terpenes have so far been identified in essential oils [7], such as functionalized derivatives of alcohols (geraniol, α-bisabolol), ketones (menthone, *p*-vetivone) of aldehydes (citronellal, sinensal), esters (γ-tepinyl acetate, cedryl acetate), and phenols (thymol).

Essential oils also contain non-terpenic compounds biogenerated by the phenylpropanoids pathway, such as eugenol, cinnamaldehyde, and safrole.

**Figure 2:**
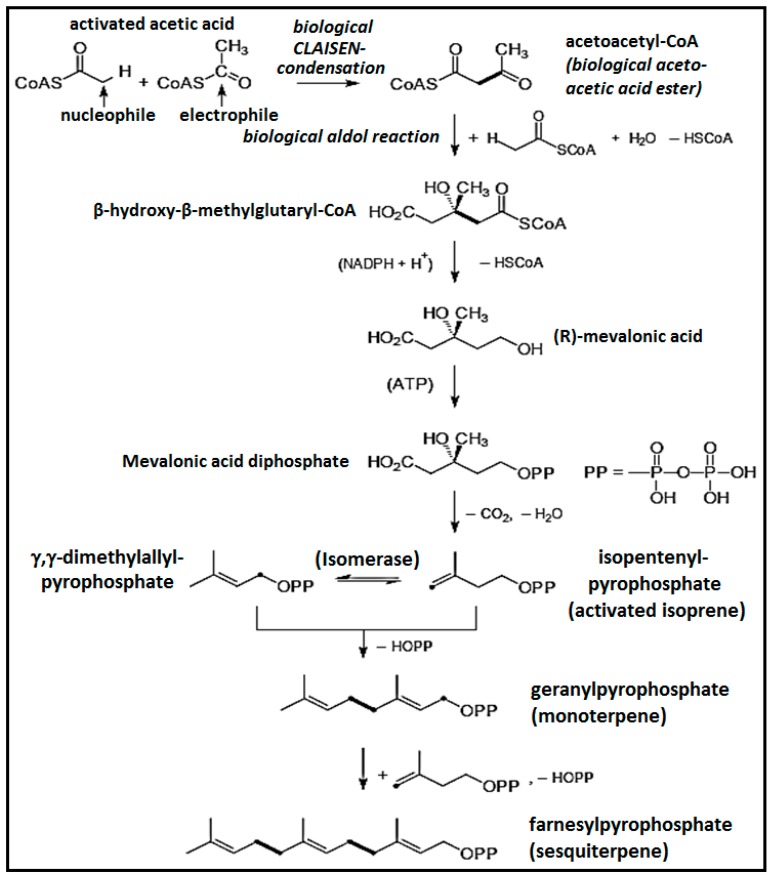
Biosynthesis pathways of monoterpenes and sesquiterpenes.

Biogenetically, terpenoids and phenylpropanoids have different primary metabolic precursors and are generated through different biosynthetic routes (Figure 2). The pathways involved in terpenoids are the mevalonate and mevalonate-independent (deoxyxylulose phosphate) pathways, whereas phenylpropanoids originate through the shikimate pathway [8,9]. Some authors have reviewed the biosynthetic pathways of terpenoids and phenylpropanoids, respectively, the enzymes and enzyme mechanisms involved, and information about genes encoding for these enzymes [8,9].

Essential oils have a very high variability of their composition, both in qualitative and quantitative terms. Various factors are responsible for this variability and can be grouped into two categories:

- Intrinsic factors related to the plant, and interaction with the environment (soil type and climate, etc.) and the maturity of the plant concerned, even at harvest time during the day,
- Extrinsic factors related to the extraction method and the environment.

The factors that determine essential oil yield and composition are numerous. In some cases, it is difficult to isolate these factors from each other as they are interrelated and influence each other. These parameters include the seasonal variations, plant organ, and degree of maturity of the plant, geographic origin, and genetics [10,11,12].

Several techniques are used for the trapping of volatiles from aromatic plants. The most often used device is the circulatory distillation apparatus described by Cocking and Middleton [13] introduced in the European Pharmacopoeia and several other pharmacopoeias. This device consists of a heated round-bottom flask into which the chopped plant material and water are placed and which is connected to a vertical condenser and a graduated tube, for the volumetric determination of the oil. At the end of the distillation process, the essential oil is separated from the water phase for further investigations. The length of distillation depends on the plant material to be investigated. It is usually fixed to 3–4 h.

A further improvement was the development of a simultaneous distillation–solvent extraction device by Likens and Nickerson in 1964 [14]. The device permits continuous concentration of volatiles during hydrodistillation in one step using a closed-circuit distillation system.

## 4. Biological Activities of Essential Oils

### 4.1. Antibacterial Activity

The antimicrobial properties of essential oils and of their constituents have been considered [15,16] and the mechanism of action has been studied in detail [17]. An important feature of essential oils are their hydrophobicity, which allows them to partition into lipids of the cell membrane of bacteria, disrupting the structure, and making it more permeable [18]. This can then cause leakage of ions and other cellular molecules [19,20,21,22]. Although a certain amount of leakage of bacterial cells can be tolerated without loss of viability, greater loss of cell contents or critical output of molecules and ions can lead to cell death [23].

EOs and/or their constituents can have a single target or multiple targets of their activity. For instance, trans-cinnamaldehyde can inhibit the growth of *Escherichia coli* and *Salmonella typhimirium* without disintegrating the OM or depleting intracellular ATP. Similar to thymol and carvacrol, trans-cinnamaldehyde likely gains access to the periplasm and deeper portions of the cell [24]. Carvone is also ineffective against the OM and does not affect the cellular ATP pool [25].

It has been reported that EOs containing mainly aldehydes or phenols, such as cinnamaldehyde, citral, carvacrol, eugenol, or thymol were characterized by the highest antibacterial activity, followed by EOs containing terpene alcohols. Other EOs, containing ketones or esters, such as β-myrcene, α-thujone, or geranyl acetate, had much weaker activity, while volatile oils containing terpene hydrocarbons were usually inactive [26,27].

Generally, essential oils characterized by a high level of phenolic compounds, such as carvacrol, eugenol, and thymol, have important antibacterial activities [17,26,28].

These compounds are responsible for the disruption of the cytoplasmic membrane, the driving force of protons, electron flow, active transport, and also coagulation of cell contents [18,23,29].

The chemical structure of essential oils affects their mode of action concerning their antibacterial activity [28]. The importance of the presence of hydroxyl group in the phenolic compounds, such as carvacrol and thymol, was confirmed [22,28,30]. However, the relative position of the phenolic hydroxyl group on the ring does not appear to influence the intensity of the antibacterial activity.

The action of thymol against *Bacillus cereus*, *Staphylococcus aureus*, and *Pseudomonas aeruginosa* appears to be comparable to that of carvacrol, for example [17,22]. However, carvacrol and thymol act differently against Gram-positive and Gram-negative species [28]. Thymol, eugenol, and carvacrol have an antimicrobial effect against a broad spectrum of bacteria: *Escherichia coli*, *Bacillus cereus*, *Listeria monocytogenes*, *Salmonella enterica*, *Clostridium jejuni*, *Lactobacillus sake*, *Staphylococcus aureus*, and *Helicobacter pyroli* [31,32]. Other families of compounds also have valuable antibacterial properties: certain alcohols, aldehydes, and ketones, monoterpene (geraniol, linalol, menthol, terpineol, thujanol, myrcenol, citronelîaî, neral, thujone, camphor, carvone, etc.), phenylpropanes (cinnamaldehyde), and monoterpenes (γ-terpinene, *p*-cymene). Among these compounds, carvacrol is the most active. Known to be non-toxic, it is used as a preservative and food flavoring in drinks, sweets, and other preparations.

It is important to mention that essential oils are more active against Gram-positive than Gram-negative bacteria [33,34,35,36,37]. The latter are less susceptible to the action of essential oils with the outer membrane surrounding the cell wall that restricts the diffusion of hydrophobic compounds through its lipopolysaccharide film [36]. Furthermore, the antibacterial activity of essential oils related to their chemical composition, the proportions of volatile molecules, and their interactions [28,33,37].

An additive effect is observed when the combination is equal to the sum of the individual effects. Antagonism is observed when the effect of one or both compounds is less important when they are tested together than when used individually [38].

A synergistic effect is observed when the combination of substances is greater than the sum of the individual effects [39]. Some studies have shown that the use of the whole essential oil provides an effect which is greater than that of the major components used together [40]. This suggests that minor components are essential for activity and may have a synergistic effect.

It has been reported additive and synergistic effects of the combinations of 1,8-cineole and aromadendrene against methicillin-resistant *Staphylococcus aureus* (MRSA) and vancomycin-resistant enterococci (VRE) and *Enterococcus faecalis* by using checkerboard and time-kill assays, respectively [41].The combined effects of plant volatile oils and benzoic acid derivatives against *L. monocytogenes* and *S. enteritidis* are considered as synergistic since the combined components allowed ≥log10 higher inhibition than the sum of the inhibitory effects of the components used separately [42]. Increased antifungal effects were caused by combinations (1:5, 1:7, and 1:9) of essential oils of *S. aromaticum* (clove) and *Rosmarinus officinalis* against *C. albicans* [43]. Moreover, Lambert et al. (2001) [17] reported that, combined, carvacrol and thymol showed additive effects against *S. aureus* and *P. aeruginosa* by using half-fold dilutions within the Bioscreen plat.

Two hypotheses have been proposed to explain synergistic effects of cinnamaldehyde/thymol or cinnamaldehyde/carvacrol against *S. typhimurium*: proving, on one hand, that thymol or carvacrol could increase the permeability of the cytoplasmic membrane, and probably enable cinnamaldehyde to be more easily transported into the cell, and, on the other hand, that thymol or carvacrol could increase the number, size, or duration of the existence of the pores created by the binding of cinnamaldehyde to proteins in the cell membrane [44]. These facts justify a synergistic effect achieved when these two components are used in combination. Mechanisms of interaction that produced antagonistic effects were less studied [45].

In addition, essential oils have also revealed to be effective on the inhibition of growth and reduction in numbers of the more serious foodborne pathogens, such as *Salmonella* spp., *E. coli* O157:H7, and *Listeria monocytogenes* [42].

### 4.2. Antioxidant Activity

Numerous studies have demonstrated the antioxidant properties of essential oils. The antioxidant potential of an essential oil depends on its composition. It is well established that phenolics and secondary metabolites with conjugated double bonds usually show substantial antioxidative properties [46]. Most of the essential oils are dominated by oxygenated monoterpenes such as alcohols (*Achillea filipendulina*), aldehydes (*Galagania fragrantissima*), ketones (*Anethum graveolens*, *Artemisia rutifolia*, *Hyssopus seravschanicus*, *Mentha longifolia*, and *Ziziphora clinopodioides*), and esters (*Salvia sclarea*). *Artemisia absinthium* and *Artemisia scoparia* predominantly contain monoterpene hydrocarbons, whereas phenolic terpenoids, such as thymol or carvacrol, characterize *Origanum tyttanthum* and *Mentha longifolia* EOs, which would explain why both plants exhibited generally the strongest antioxidant activity. Thymol and carvacrol, which are predominant in *Origanum tyttanthum*, are also responsible for the antioxidant activity of several other essential oils, such as *Mentha longifolia* and *Thymus serpyllus* [47].

The essential oils of cinnamon, nutmeg, clove, basil, parsley, oregano, and thyme are characterized by the most important antioxidant properties [43]. Thymol and carvacrol are the most active compounds. Their activity is related to their phenolic structure. These phenolic compounds have redox properties and, thus, play an important role in neutralizing free radicals and also in peroxide decomposition [40]. The antioxidant activity of essential oils is also due to certain alcohols, ethers, ketones, aldehydes, and monoterpenes: linalool, 1,8-CineoIe, geranial/neral, citronellal, isomenthone, menthone, and some monoterpenes: α-Terpinene, β-Terpinene and α-Terpinolene [43].

Essential oils with important scavenging capacity of free radicals may play an important role in some disease prevention, such as brain dysfunction, cancer, heart disease, and immune system decline. In fact, these diseases may result from cellular damage caused by free radicals [43,44].

EOs have shown their action as hepatoprotective agents in ageing polyunsaturated fatty acids mammals and it has been proved that they possess a beneficial impact upon the PUFAs, in particular the long chain C20 and C22 acids [48]. Moreover, essential oils being able to scavenge free radicals may also play an important role in some disease prevention, such as brain dysfunction, cancer, heart disease, and immune system decline [49].

Sharififar et al. (2011) [50] evaluated the antioxidant activity of *Zataria multiflora* Boiss. (Lamiaceae) essential oil in rats. Antioxidant activity was measured by the test of 1,1-diphenyl-2-picrylhydrazyl (DPPH) radical inhibition and inhibition of lipid peroxidation by measuring the index of thiobarbituric acid reactive substances (TBARs). Three doses of 100, 200, and 400 μL/kg were administered to animals by intra gastric intubation (i.g) routh for 10 days. The blood was collected in eleventh day through direct puncture and the liver was rapidly excised. The histopathology studies of the animals were compared to animals in butylated hydroxyl toluene (BHT) group. The authors reported that all *Zataria multiflora* oils ZMO tested doses were able to scavenge DPPH radical (*p* < 0.05). Moreover, ZMO decreased TBARs in a dose-dependent manner. No alteration in liver function test LFT enzymes or changes in histopathology of the liver was considered in ZMO treated groups. The results indicated that ZMO might be used in human healthy and food industry.

According to Manjamalai and Grace [51], essential oil of *Wedelia chinensis* (Osbeck) increases both the level of catalase and glutathione peroxidase in the lung and liver tissues, whereas in the serum the level of catalase decreased on the 22nd day (2.32 ± 0.016 Lung tissue 6.47 ± 0.060 liver tissue, 0.94 ± 0.007 serum). Furthermore, the level of Glutathione Peroxidase GPx in the liver (the range) was found to be decreased in the EO-treated group compared to the cancer-induced group and control group, whereas the level of GPx in the lung tissue was found to be low (76.2 ± 1.66).

### 4.3. Anti-Inflammatory Activity

Inflammation is a normal protective response induced by tissue injury or infection and functions to combat invaders in the body (microorganisms and non-self cells) and to remove dead or damaged host cells. The inflammatory response induces an increase of permeability of endothelial lining cells and influxes of blood leukocytes into the interstitium, oxidative burst, and release of cytokines, such as interleukins and tumor necrosis factor-α (TNF-α). It also stimulates the activity of several enzymes (oxygenases, nitric oxide synthases, peroxidases, etc.), as well as the arachidonic acid metabolism. Recently, essential oils have been used in clinical settings to treat inflammatory diseases, such as rheumatism, allergies, or arthritis [45]. *Melaleuca alternifolia* EO was reported to have a considerable anti-inflammatory activity [46,47,48].This activity is correlated with its major compound: α-terpineol [49]. The active compounds act by inhibiting the release of histamine or reducing the production of inflammation mediators. Geranium essential oil is another example [45]. Linalool and linalyl acetate showed anti-inflammatory activity on oedema of paw-induced mouse carrageenan [50]. Yoon et al. [52] reported that the oils of *Torreya nucifera* Siebold et Zucc. oil, mainly constituted by limonene, δ-3-carene, and α-pinene, have an inhibitory effect on COX-2, thus inducing a significant inhibitory effect on prostaglandin (PGE2) production. Furthermore, 1,8-cineole, present in many essential oils, was reported as an inhibitor of leukotrienes (LTB4) and PGE2, biogenerated both from pathways of arachidonic acid metabolism [52].

The anti-inflammatory activity of essential oils may be attributed not only to their antioxidant activities but also to their interactions with signaling cascades involving cytokines and regulatory transcription factors, and on the expression of pro-inflammatory genes. Essential oils, therefore, represent a new option in the treatment of inflammatory diseases.

### 4.4. Cancer Chemoprotective Activity

The varied therapeutic potential of essential oils attracted, in recent years, the attention of researchers for their potential activity against cancer. They and their volatile constituents of the studies target the discovery of new anticancer natural products [41]. Essential oils would act in the prevention of cancer, as well as at its removal. It is well known that certain foods, such as garlic and turmeric, are good sources of anticancer agents [53]. Garlic essential oil is a source of sulfur compounds recognized for their preventive effect against cancer [54,55]. Diallylsulfide, diallyldisulfide, and diallyltrisulfide are examples. According to Wu et al. [56], these compounds activate, in rats, the enzymes involved in the detoxification process of hepatic phase 1 (disintegration of chemical bonds that link carcinogenic toxins to each other) and phase 2 (bonds to toxins released detoxifying enzymes, such as glutathione *S*-transferase).

Metabolism happens mainly in the liver, the body’s largest internal organ. The portal vein carries blood from the small intestine directly to the liver. Sixty percent of liver tissue is made up of hepatic cells. More chemical processes happen in these than in any other group of cells in the body. Phase 1 metabolism involves chemical reactions, such as oxidation (most common), reduction, and hydrolysis. There are three possible results of phase 1 metabolism. The drug becomes completely inactive. In other words, the metabolites are pharmacologically inactive. One or more of the metabolites are pharmacologically active, but less so than the original drug. The original substance is not pharmacologically active, but one of its metabolites is. The original substance is called a prodrug.

Phase 2 metabolism involves reactions that chemically change the drug or phase 1 metabolites into compounds that are soluble enough to be excreted in urine. In these reactions, the molecule (drug or metabolite) is attached to an ionisable grouping. This is called conjugation and the product is called a conjugate. Metabolites formed in phase 2 are unlikely to be pharmacologically active. Some drugs undergo either phase 1 or phase 2 metabolism, but most undergo phase 1 metabolism followed by phase 2 metabolism.

Another example is myristicin, an allylbenzene present on a certain essential oil, especially that of nutmeg (*Myristica fragrans*). This molecule is known to activate glutathione *S*-transferase in mice [57] and inhibit carcinogenesis induced by benzo(a)pyrene in the lungs of mice [58]. Recently, it has been discovered that myristicin induces apoptosis in neuroblastoma (SK-N-SH) in humans [58]. There are other volatile compounds that showed a cytotoxic activity against various cancer cell lines [43]. Geraniol decreases the resistance of colon cancer cells (TC118) to 5-fluorouracil, an anticancer agent. Therefore, geraniol enhances this inhibitory effect of tumour growth 5-fluorouracil [59,60]. The essential oil of balsam fir and α-Humulene, showed significant anticancer activity in several cell lines and low toxicity to healthy cells [61].

In addition, anticancer activity of d-limonene, the main component of Citrus essential oil has been proven, especially at the level of stomach cancer and liver [62]. The α-Bisabolol, an abundant sesquiterpene alcohol in chamomile essential oil (*Matricaria*), has an antigliomale activity [63]. Many essential oils have a cytotoxic activity namely *Melissa officinalis* [64], *Melaleuca alternifolia* [65], *Artemisia annua* [66], and *Comptonia peregrina* [67].

### 4.5. Cytotoxicity

Due to their complex chemical composition, essential oils have no specific cellular ligands [21]. As lipophilic mixtures, they are able to cross the cell membrane and degrade the layers of polysaccharides, phospholipids and fatty acids, and permeabilize. This cytotoxicity appears to include such membrane damage. In bacteria, the membrane permeabilization is associated with the loss of ions and the reduction of the membrane potential, the collapse of the proton pump and the depletion of the ATP pool [22,68,69,70]. Essential oils may coagulate the cytoplasm [17] and damage lipids and proteins [22,40]. Damage to the wall and the cell membrane can lead to the leakage of macromolecules and lysis [17,20,71].

In addition, essential oils change membrane fluidity, which becomes abnormally permeable, resulting in a leakage of radicals, cytochrome C, the Ca^2+^ ions, and proteins, like in the case of oxidative stress. This permeabilization of the outer and inner membranes causes cell death by apoptosis and necrosis [72,73]. Ultrastructural alteration of the cell can be observed at a plurality of compartments [52,74,75]. The interruption of the viral envelope herpes simplex virus HSV by essential oils can also be observed by electron microscopy [76]. The induction of membrane damage was also confirmed by an analysis showing that microtubule *Saccharomyces cerevisiae* genes involved in the biosynthesis of ergosterol, the absorption of sterols, lipid metabolism, the structure and function of cell wall cellular detoxification, and transport are affected by treatment with α-terpinene [77].

Recent work on the yeast *Saccharomyces cerevisiae*, has shown that the cytotoxicity of some essential oils based on the ability to form colonies differs significantly in relation to their chemical composition. Generally, essential oil cytotoxicity mainly correlates to the presence of phenols, alcohols, and monoterpene aldehydes [78,79]. The cytotoxic properties of essential oils are of great importance because they assume their use not only against certain human pathogens and animal parasites, but also in the preservation of agricultural and marine products against microbial attack. Indeed, some components of essential oils are effective against a variety of microorganisms as bacteria [80], viruses [81], fungi [77,82,83,84], protozoa [85], parasites [86,87,88], mites, and others.

In addition, α-humulene shows cytotoxicity against breast cancer cells in vitro. α-humulene was reported to be responsible for cytotoxicity (CI_50_ 55 mM) [89]. It induced a dose- and time-dependent decrease in cellular glutathione (GSH) content and an increase in reactive oxygen species (ROS) production.

Furthermore, Zeytinoglu et al. [90], focusing on the effects of carvacrol, one of the main compounds in the EO of oregano, on the DNA synthesis of *N*-ras transformed mouse myoblast CO25 cells, finding that this monoterpenic phenol was able to inhibit the DNA synthesis in the growth medium and ras-activating medium, which contained dexamethasone. They proposed that it may be valuable in cancer therapy because of its growth inhibition of myoblast cells, even after activation of mutated *N*-ras-oncogene.

The EO of the Anonaceae *Xylopia aethiopica* (Ethiopian pepper), a plant grown in Nigeria, showed, at a concentration of 5 mg/mL, a cytotoxic effect in the carcinoma cell line (Hep-2) [91].

Moreover, Yu et al. [92] tested the essential oil of the rhizome of the *Aristolochiaceae Aristolochia mollissima* for its cytotoxicity on four human cancer cell lines (ACHN, Bel-7402, Hep G2, HeLa). The rhizome oil possessed a significantly greater cytotoxic effect on these cell lines than the oil extracted from the aerial plant.

Linalool inhibited only moderate cell proliferation; however, in subtoxic concentrations potentiates doxorubicin-induced cytotoxicity and proapoptotic effects in both cell lines, MCF7 WT and MCF7 AdrR. This monoterpene improves the therapeutic index in the management of breast cancer, especially multidrug resistance (MDR) tumors [93].

An in vitro cytotoxicity assay indicated that the EO of *Cyperus rotundus* (Cyperaceae) characterized by the predominance of cyperene, α-Cyperone, isolongifolen-5-one, rotundene, and cyperorotundene, was very effective against L1210 leukemia cells, which correlates with significantly increased apoptotic DNA fragmentation [94].

### 4.6. Allelopathic Activity

According to the International Allelopathy Society (IAS), allelopathy was defined in 1996 as “The science that studies any process involving secondary metabolites produced by plants, algae, bacteria and fungi that influences the growth and development of agricultural and biological systems”. Allelopathic interactions derive from the production of secondary metabolites. The secondary metabolites are synthesized for a wide range defense by plant and microorganisms. The secondary metabolites involved are called allelochemicals [95].

Volatile oils and their constituents are being explored for weed and pest management, and are viewed as an important source of lead molecules in agriculture [96]. Bioactive terpenoids constitute an important part of the defensive mechanisms of a large number of organisms and represent a fairly untapped source of active compounds of potential use both in the agricultural field [97]. In fact, a large number of highly phytotoxic allelochemicals are derived from the terpenoid pathway [98] and the phytotoxicity of essential oils has been investigated [98,99,100,101]. The allelopathic activity of *Melaleuca alternifolia* (Maiden and Betche) Cheel (tea tree) essential oil was investigated by Angelini et al., [101] against *Trichoderma harzianum*, which is a fungal contaminant that causes extensive losses in the cultivation of *Pleurotus* species. This essential oil has, in vitro, an allelopathic ability to control *Trichoderma harzianum*. The antifungal activity of *M. alternifolia* essential oil and antagonist activities between *Pleurotus* species against three *T. Harzianum* strains were studied in dual-culture experiments done with different concentrations.

Santos et al. [102] reported that leaves’ and rhizomes’ EOs caused a decrease in dry matter. They also reported a reduction of shoot length in lettuce seedlings. Evaluating the effect of these EOs on the germination and vigor of the lettuce seedlings, they noticed a reduction of these parameters and concluded that rhizomes’ oil caused a greater reduction in all of the variables than the oil from the leaves.

*Portulaca oleracea* seeds’ germination and growth were significantly decreased by the treatment with rosemary EO [103]. These authors reported that a concentration of 1000 ppm of this oil, rosemary decreased *Portulaca oleracea* seed germination to 76 percent. They also noted that Artemisia and lavender essential oils have strong allelopathic effects and prevents weed germination and growth of *Portulaca oleracea*, which would be a promising result in the organic cultivation of crops to be followed, and it can be used in the production of herbicides with natural origin.

Furthermore, de Oliveira et al. [104] reported that *Callistemon viminalis* EO affected the growth of lettuce seedlings and caused a reduction in the length of shoots and the root system. This reduction was proportional to the EO concentration.

The results of the research of Saad and Abdelgaleil [105] revealed a correlation between EOs chemical composition and their effects on germination and seedling growth. It was reported that the most active compounds belonged to the groups of ketones and alcohols and were followed by the group of aldehydes and phenols [106]. Moreover, Kotan et al. [107] suggested that, in general, a potent phytotoxic activity of plant EOs is correlated to a high amount of oxygenated monoterpenes.

Almost all the effective oils had high percentages of oxygenated monoterpenes and this was in agreement with previous work of de Almeida et al. and Vokou et al. [108,109].

Dudai et al. [103] reported that monoterpenes act on seeds at very low levels. In particular, among the Lamiaceae family, many species release phytotoxic monoterpenes that hinder the development of herbaceous species, including pinene, limonene, *p*-Cymene, and 1,8-cineole [101]. Moreover, it is well known that monoterpenes in the essential oils have phytotoxic effects that may cause anatomical and physiological changes in plant seedlings leading to accumulation of lipid globules in the cytoplasm, reduction in some organelles such as mitochondria, possibly due to inhibition of DNA synthesis or disruption of membranes surrounding mitochondria and nuclei [110,111]. Since the continued use of synthetic herbicides may threaten sustainable agricultural production and result in serious ecological and environmental problems, essential oils with allelopatic properties could be exploited as in alternative strategies leading to the development of biodegradable and non-toxic compounds [112].

### 4.7. Repellent and Insecticidal Activity

Essential oils constitute a rich bank of structurally-diverse compounds with a variety of insecticidal and repellent mechanisms. Numerous studies have demonstrated that these compounds, as well as their parent blends, possess biological activity capable of eliciting adverse effects in arthropod pests. Several factors affecting the commercialization of plant essential oil extracts as repellents include regulatory requirements, intellectual property value, biological activity, product performance, and product quality [113].

The toxic effect of essential oils was not only suitable for granary insects but also for flying insects: *Gaultheria* (Ericaceae) and *Eucalyptus* (Myrtaceae) oils exhibited very high killing power on insects, such as the rice weevil *Sitophilus oryzae*, the beetles *Callosobruchus chinensis* (Coleoptera: Bruchidae) and *S. paniceum*, and also on *M. domestica* [114]. Actually, the activities of essential oils on species are manifold. *Mentha, Lavandula* (Lamiaceae), or *Pinus* (Pinaceae) essential oils were noted for their toxicity against *Myzus persicae* (Homoptera: Aphididae) and the greenhouse white fly *Trialeurodes vaporariorum* (Homoptera: Aleyrodidae), as well as the Colorado beetle *Leptinotarsa decemlineata* (Coleoptera: Chrysomelidae) and the pear bug *Stephanitis pyri* (Hymenoptera: Stephanidae) [115].

Commonly, essential oils can be inhaled, ingested, or skin-absorbed by insects. The fumigant toxicity of essential oils and their main components, the volatile monoterpenes, has been described [116]. Insects were also very sensitive to topical applications *Sitophilus zea-mais* (Coleoptera: Curculionidae), *Tribolium castaneum* and *Prostephanus truncatus* (Coleoptera: Bostrychidae) reacted to citrus (Rutacae) essential oils. *Pediculus capitis* (Anoplura: Pediculidae), *Anopheles funestus* (Diptera: Culicidae), *Cimex lectularius* (Hemiptera: Cimicidae), and *Periplaneta orientalis* (Dictyoptera: Blattidae) were killed by contact with *Eucalyptus saligna* (Myrtaceae) oil within 2 to 30 min.

Essential oils belonging to plants in the citronella genus (*Poaceae*) are commonly used as ingredients of plant-based mosquito repellents, mainly *Cymbopogon nardus*, which is sold in Europe and North America in commercial preparations [117].

## 5. Conclusions

Thanks to their numerous biological activities, essential oils have to be valorized via several domains, mainly human health, green chemistry, and sustainable agriculture. However, numerous investigations should be carried out on their mode of action and their probable toxicological effects in order to optimize their potential uses.

## References

[1] Guenther E. (1948). The Essential Oils.

[2] Association Française de Normalisation (AFNOR) (2000). Huiles Essentielles, Tome 2, Monographies Relatives Aux Huiles Essentielles.

[3] Carette Delacour A.S. (2000). La Lavande et son Huile Essentielle. Ph.D. Thesis.

[4] Sell C.S. (2006). The Chemistry of Fragrance. From Perfumer to Consumer.

[5] Vainstein A., Lewinsohn E., Pichersky E., Weiss D. (2001). Floral Fragrance. New Inroads into an Old Commodity. Plant Physiol..

[6] Pophof B., Stange G., Abrell L. (2005). Volatile Organic Compounds as Signals in a Plant—Herbivore System: Electrophysiological Responses in Olfactory Sensilla of the Moth *Cactoblastis cactorum*. Chem. Senses.

[7] Modzelewska A., Sur S., Kumar K.S., Khan S.R. (2005). Sesquiterpenes: Natural products that decrease cancer growth. Curr. Med. Chem. Anti-Cancer Agents.

[8] Litchenthaler H.K. (1999). The 1-deoxy-d-xylulose-5-phosphate pathway of isoprenoid biosynthesis in plants. Annu. Rev. Plant Physiol. Plant Mol. Biol..

[9] Dewick P.M. (2002). The biosynthesis of C5-C-25 terpenoid components. Nat. Prod. Rep..

[10] Marotti M., Piccaglia R., Giovanelli E. (1994). Effects of variety and ontogenic stage on the essential oil composition and biological activity of fennel (*Foeniculum vulgare* Mill.). J. Essent. Oil Res..

[11] Hussain A.I., Anwar F., Sherazi S.T.H., Przybylski R. (2008). Chemical composition, antioxidant and antimicrobial activities of basil (*Ocimum basilicum*) essential oils depends on seasonal variations. Food Chem..

[12] Anwar F., Hussain A.I., Sherazi S.T.H., Bhanger M.I. (2009). Changes in composition and antioxidant and antimicrobial activities of essential oil of fennel (*Foeniculum vulgare* Mill.) fruit at different stages of maturity. J. Herbs Spices Med. Plants.

[13] Cocking T.T., Middleton G. (1935). Improved method for the estimation of the essential oil content of drugs. Q. J. Pharm. Pharmacol..

[14] Nickerson G., Likens S. (1996). Gas chromatographic evidence for the occurrence of hop oil components in beer. J. Chromatogr..

[15] Shelef L.A. (1983). Antimicrobial effects of spices. J. Food Saf..

[16] Nychas G.J.E., Gould G.W. (1995). Natural antimicrobials from plants. New Methods of Food Preservation.

[17] Lambert R.J.W., Skandamis P.N., Coote P., Nychas G.J.E. (2001). A study of the minimum inhibitory concentration and mode of action of oregano essential oil, thymol and carvacrol. J. Appl. Microbiol..

[18] Sikkema J., de Bont J.A.M., Poolman B. (1994). Interactions of cyclic hydrocarbons with biological membranes. J. Biol. Chem..

[19] Gustafson J.E., Liew Y.C., Chew S., Markham J.L., Bell H.C., Wyllie S.G., Warmington J.R. (1998). Effects of tea tree oil on *Escherichia coli*. Lett. Appl. Microbiol..

[20] Cox S.D., Mann C.M., Markham J.L., Bell H.C., Gustafson J.E., Warmington J.R., Wyllie S.G. (2000). The mode of antimicrobial action of essential oil of *Melaleuca alternifola* (tea tree oil). J. Appl. Microbiol..

[21] Carson C.F., Riley T.V. (1995). Antimicrobial activity of the major components of the essential oil of *Melaleuca alternifolia*. J. Appl. Bacteriol..

[22] Ultee A., Bennink M.H.J., Moezelaar R. (2002). The phenolic hydroxyl group of carvacrol is essential for action against the food-borne pathogen *Bacillus cereus*. Appl. Environ. Microbiol..

[23] Denyer S.P., Hugo W.B., Denyer S.P., Hugo W.B. (1991). Biocide-induced damage to the bacterial cytoplasmic membrane. Mechanisms of Action of Chemical Biocides, the Society for Applied Bacteriology, Technical Series No 27.

[24] Farag R.S., Daw Z.Y., Hewedi F.M., El-Baroty G.S.A. (1989). Antimicrobial activity of some Egyptian spice essential oils. J. Food Prot..

[25] Cosentino S., Tuberoso C.I.G., Pisano B., Satta M., Mascia V., Arzedi E., Palmas F. (2002). In vitro antimicrobial activity and chemical composition of Sardinian Thymus essential oils. Lett. Appl. Microbiol..

[26] Dorman H.J.D., Deans S.G. (2000). Antimicrobial agents from plants: Antibacterial activity of plant volatile oils. J. Appl. Microbiol..

[27] Davidson P.M., Doyle M.P., Beuchat L.R., Montville T.J. (1997). Chemical preservatives and natural antimicrobial compounds. Food Microbiology: Fundamentals and Frontiers.

[28] Knobloch K., Weigand H., Weis N., Schwarm H.M., Vigenschow H., Brunke E.J. (1986). Action of terpenoids on energy metabolism. Progress in Essential Oil Research: 16th International Symposium on Essential Oils.

[29] Pauli A. (2001). Antimicrobial properties of essential oil constituents. Int. J. Aromather..

[30] Fabian D., Sabol M., Domaracké K., Bujnékovâ D. (2006). Essential oils, their antimicrobial activity against *Escherichia coli* and effect on intestinal cell viability. Toxicol. in Vitro.

[31] Marino M., Bersani C., Comi G. (1999). Antimicrobial activity of the essential oils of *Thymus vulgaris* L. measured using a bioimpedometric method. J. Food Prot..

[32] Senatore F., Napolitano F., Ozcan M. (2000). Composition and antibacterial activity of the essential oil from *Crithmum maritimum* L. (Apiaceae) growing wild in Turkey. Flav. Frag. J..

[33] Canillac N., Mourey A. (2001). Antibacterial activity of the essential oil of *Picea excelsa* on *Listeria*, *Staphylococcus aureus* and coliform bacteria. Food Microbiol..

[34] Cimanga K., Kambu K., Tona L., Apers S., de Bruyne T., Hermans N., Totté J., Pieters L., Vlietinck A.J. (2002). Correlation between chemical composition and antibacterial activity of essential oils of some aromatic medicinal plants growing in the Democratic Republic of Congo. J. Ethnopharmacol..

[35] Delaquis P.J., Stanich K., Girard B., Mazza G. (2002). Antimicrobial activity of individual and mixed fractions of dill, cilantro, coriander and eucalyptus essential oils. Int. J. Food Microbiol..

[36] Ratledge C., Wilkinson S.G., Ratledge C., Wilkinson S.G. (1988). An overview of microbial lipids. Microbial Lipids.

[37] Davidson P.M., Parish M.E. (1989). Methods for testing the efficacy of food antimicrobials. Food Technol..

[38] Gill A.O., Delaquis P., Russo P., Holley R.A. (2002). Evaluation of antilisterial action of cilantro oil on vacuum packed ham. Int. J. Food Microbiol..

[39] Reichling J., Schnitzler P., Suschke U., Saller R. (2009). Essential oils of aromatic plants with antibacterial, antifungal, antiviral, and cytotocic properties-an overview. Forsch. Komplement..

[40] Burt S. (2004). Essential oils: Their antibacterial properties and potential applications in foods. Int. J. Food Microbiol..

[41] Edris A.E. (2007). Pharmaceutical and Therapeutic Potentials of Essential Oils and Their Individual Volatile Constituents. Phytother. Res..

[42] Braga P.C., dal Sasso M., Culici M., Gasastri L., Marceca M.X., Guffanti E.E. (2006). Antioxidant potential of thymol determined by chemiluminescence inhibition in human neutrophils and cell-free systems. Pharmacology.

[43] Aruoma O.I. (1998). Free radicals, oxidative stress, and antioxidants in human health and disease. J. Am. Oil Chem. Soc..

[44] Kamatou G.P.P., Viljoen A.M. (2010). A review of the application and pharmacological properties of α-Bisabolol and α-Bisabolol-rich oils. J. Am. Oil Chem. Soc..

[45] Maruyama N., Sekimoto N., Ishibashi H. (2005). Suppression of neutrophil accumulation in mice by cutaneous application of geranium essential oil. J. Inflamm..

[46] Koh K.J., Pearce A.L., Marshman G., Finlay-Jones J.J., Hart P.H. (2002). Tea tree oil reduces histamine-induced skin inflammation. Br. J. Dermatol..

[47] Caldefie-Chézet F., Guerry M., Chalchat J.C., Fusillier C., Vasson M.P., Guillot J. (2004). Antiinflammatory effects of *Malaleuca alternifolia* essential oil on human polymorphonuciear neutrophils and monocytes. Free Radic. Res..

[48] Caldefie-Chézet F., Fusillier C., Jarde T., Laroye H., Damez M., Vasson M.P. (2006). Potential antiinflammatory effects of *Malaleuca alternifolia* essential oil on human peripheral blood leukocytes. Phytother. Res..

[49] Hart P.H., Brand C., Carson C.F., Riley T.V., Prager R.H., Finlay-Jones J.J. (2000). Terpinen-4-ol, the main component of the essential oil of *Malaleuca altemifolia* (tea tree oil), suppresses inflammatory mediator production by activated human monocytes. Inflamm. Res..

[50] Sharififar F., Mirtajadini M., Azampour M.J., Zamani E. (2012). Essential Oil and Methanolic Extract of *Zataria multiflora* Boiss with Anticholinesterase Effect. Pak. J. Biol. Sci..

[51] Manjamalai A., Jiflin G.J., Grace V.M. (2012). Study on the effect of essential oil of *Wedelia chinensis* (Osbeck) against microbes and inflammation. Asian J. Pharm. Clin. Res..

[52] Yoon H.S., Moon S.C., Kim N.D., Park B.S., Jeong M.H., Yoo Y.H. (2000). Genistein induces apoptosis of RPE-J cells by opening mitochondrial PTP. Biochem. Biophys. Res. Commun..

[53] Pyun M.S., Shin S. (2006). Antifungal effects of the volatile oils from Asiium plants against *Trichophyton* species and synergism of the oils wih ketoconazole. Phytomedicine.

[54] Milner J.A. (2001). A historical perspective on garlic and cancer. Recent advances on the nutritional effects associated with the use of garlic as a supplement. J. Nutr..

[55] Milner J.A. (2006). Preclinical perspectives on garlic and cancer. Signifiance of garlic and its constituents in cancer and cardiovascular disease. J. Nutr..

[56] Wu C.C., Sheen L.Y., Chen H.W., Kuo W.W., Tsai S.J., Lii C.K. (2002). Differential effects of garlic oil and its three major organosulfur components on the hepatic detoxification system in rats. J. Agric. Food Chem..

[57] Ahmad H., Tijerina M.T., Tobola A.S. (1997). Preferential overexpression of a class MU glutathione *S*-transferase subunit in mouse liver by myristicin. Biochem. Biophys. Res. Commun..

[58] Zheng G., Kenny P., Lam L. (1992). Inhibition of benzo[α]-pyrene-induced tumorigenesis by myristicin, a volatile aroma constituent of parsley leaf oil. Carcinogenesis.

[59] Lee B.K., Kim J.H., Jung J.W., Choi J.W., Han E.S., Lee S.H., Ko K.H., Ryu J.H. (2005). Myristicin induced neurotoxicity in human neuroblastoma MSK-N-SH cells. Toxicol. Lett..

[60] Carnesecchi S., Bras-Gonçalves R., Bradaia A., Zeisel M., Gossé F., Poupon M.F., Raul F. (2004). Geraniol, a component of plant essential oils, modulates DNA synthesis and potentiates 5-fluorourcil efficacy on human colon tumor xenografts. Cancer Lett..

[61] Carnesecchi S., Langley K., Exinger F., Gossé F., Raul F. (2002). Geraniol, a component of plant essential oils, sensitizes human colonie cancer cells to 5-fluorouracil treatment. J. Pharm. Exp. Ther..

[62] Legault J., Dahl W., Debiton E., Pichette A., Madelmont J.C. (2003). Antitumor activity of balsam fir oil: Production of reactive oxygen species induced by α-Humulene as possible mechanism of action. Planta Med..

[63] Uedo N., Tatsuta M., Lishi H., Baba M., Sakai N., Yano H., Otani T. (1999). Inhibition by Dlimonene of gastric carcinogenesis induced by *N* methyl *N*’ nitro *N*-nitrosoguanimidine in wistar rats. Cancer Lett..

[64] Cavalieri E., Mariotto S., Fabrizi C., Carcereri de Prati A., Gottardo R., Leone S., Berra L.V., Lauro G.M., Ciampa A.R., Suzuki H. (2004). α-Bisabolol, a nontoxic natural compound, strongly induces apoptosis in glioma cells. Biochem. Biophys. Res. Commun..

[65] De Sousa A., Alviano A., Blank A., Alves P., Alviano C., Gattass C. (2004). *Melisa officinalis* L. essential oil: Antitumoral and antioxidant activities. J. Pharm. Pharmacol..

[66] Calcabrini A., Stringaro A., Toccacieli L., Meschini S., Marra M., Colone M., Salvatore G., MondeIlo F., Arancia G., Molinari A. (2004). Terpinen-4-ol, the main component of *Melaieuca aitemifolia* (tea tree) oil inhibits the in vitro growth of human melanoma cells. J. Investig. Dermatol..

[67] Li Y., Li M., Wang L., Jiang Z., Li W., Li H. (2004). Induction of apoptosis of cultured hepatocarcinoma cell by essential oil of *Artemisia annua* L.. Sichuan Da Xue Xue Bao Yi Xue Ban.

[68] Sylvestre M., Pichette A., Lavoie S., Longtin A., Legault J. (2007). Composition and cytotoxic activity of the leaf essential oil of *Comptonia peregrine* L. Coulter. Phytother. Res..

[69] Ultee A., Kets E.P., Alberda M., Hoekstra F.A., Smid E.J. (2000). Adaptation of the food-borne pathogen Bacillus cereus to carvacrol. Arch. Microbiol..

[70] Di Pasqua R., Hoskins N., Betts G., Mauriello G. (2006). Changes in membrane fatty acids composition of microbial cells induced by addiction of thymol, carvacrol, limonene, cinnamaldehyde, and eugenol in the growing media. J. Agric. Food Chem..

[71] Turina A.V., Nolan M.V., Zygadlo J.A., Perillo M.A. (2006). Natural terpenes: Self-assembly and membrane partitioning. Biophys. Chem..

[72] Oussalah M., Caillet S., Lacroix M. (2006). Mechanism of action of Spanish oregano, Chinese cinnamon, and savory essential oils against cell membranes and walls of *Escherichia coli* O157:H7 and *Listeria monocytogenes*. J. Food Prot..

[73] Novgorodov S.A., Gudz T.I. (1996). Permeability transition pore of the inner mitochondrial membrane can operate in two open states with different selectivities. J. Bioenerg. Biomembr..

[74] Vercesi A.E., Kowaltowski A.J., Grijalba M.T., Meinicke A.R., Castilho R.F. (1997). The role of reactive oxygen species in mitochondrial permeability transition. Biosci. Rep..

[75] Armstrong J.S. (2006). Mitochondrial membrane permeabilization: The sine qua non for cell death. Bioessays.

[76] Soylu E.M., Soylu S., Kurt S. (2006). Antimicrobial activity of the essential oils of various plants against tomato late blight disease agent *Phytophthora infestans*. Mycopathologia.

[77] Santoro G.F., Cardoso M.G., Guimaraes L.G., Mendonca L.Z., Soares M.J. (2007). Trypanosoma cruzi: Activity of essential oils from *Achillea millefolium* L., *Syzygiumaromaticum* L. and *Ocimumbasilicum* L. on epimastigotes and trypomastigotes. Exp. Parasitol..

[78] Santoro G.F., Das Gracas Cardoso M., Guimaraes L.G., Salgado A.P., Menna-Barreto R.F., Soares M.J. (2007). Effect of Oregano (*Origanum vulgare* L.) and Thyme (*Thymus vulgaris* L.) essential oils on *Trypanosoma cruzi* (Protozoa: Kinetoplastida) growth and ultrastructure. Parasitol. Res..

[79] Schnitzler P., Koch C., Reichling J. (2007). Susceptibility of drugresistant clinical HSV-1 strains to essential oils of Ginger, Thyme, Hyssop and Sandalwood. Antimicrob. Agents Chemother..

[80] Parveen M., Hasan M.K., Takahashi J., Murata Y., Kitagawa E., Kodama O., Iwahashi H. (2004). Response of *Saccharomyces cerevisiae* to a monoterpene: Evaluation of antifungal potential by DNA microarray analysis. J. Antimicrob. Chemother..

[81] Hong E.J., Na K.J., Choi I.G., Choi K.C., Jeung E.B. (2004). Antibacterial and antifungal effects of essential oils from coniferous trees. Biol. Pharm. Bull..

[82] Rota C., Carraminana J.J., Burillo J., Herrera A. (2004). In vitro antimicrobial activity of essential oils from aromatic plants against selected foodborne pathogens. J. Food Prot..

[83] Si W., Gong J., Tsao R., Zhou T., Yu H., Poppe C., Johnson R., Du Z. (2006). Antimicrobial activity of essential oils and structurally related synthetic food additives towards selected pathogenic and beneficial gut bacteria. J. Appl. Microbiol..

[84] Sonboli A., Babakhani B., Mehrabian A.R. (2006). Antimicrobial activity of six constituents of essential oil from Salvia. Z. Naturforschung.

[85] Bruni R., Medici A., Andreotti E., Fantin C., Muzzoli M., Dehesa M. (2003). Chemical composition and biological activities of Isphingo essential oil, a traditional Ecuadorian spice from *Ocotea quixos* (Lam.) Kosterm. (Lauraceae) flower calices. Food Chem..

[86] Sacchetti G., Maietti S., Muzzoli M., Scaglianti M., Manfredini S., Radice M., Bruni R. (2005). Comparative evaluation of 11 essential oils of different origin as functional antioxidants, antiradicals and antimicrobials in food. Food Chem..

[87] Basile A., Senatore F., Gargano R., Sorbo S., Del Pezzo M., Lavitola A., Ritieni A., Bruno M., Spatuzzi D., Rigano D. (2006). Antibacterial and antioxidant activities in *Sideritis italica* (Miller) Greuter et Burdet essential oils. J. Ethnopharmacol..

[88] Duschatzky C.B., Possetto M.L., Talarico L.B., Garcia C.C., Michis F., Almeida N.V., de Lampasona M.P., Schuff C., Damonte E.B. (2005). Evaluation of chemical and antiviral properties of essential oils from South American plants. Antivir. Chem. Chemother..

[89] El Hadri A., Gómez Del Río M.A., Sanz J., González Coloma A., Idaomar M., Ribas Ozonas B., Benedí González J., Sánchez Reus M.I. (2010). Cytotoxic activity of α-humulene and transcaryophyllene from *Salvia officinalis* in animal and human tumor cells. An. Real Acad. Nac. Farm..

[90] Zeytinoglu H., Incesu Z., Baser K.H. (2003). Inhibition of DNA synthesis by carvacrol in mouse myoblast cells bearing a human NRAS oncogene. Phytomedicine.

[91] Asekun O.T., Adeniyi B.A. (2004). Antimicrobial and cytotoxic activities of the fruit essential oil of *Xylopia aethiopica* from Nigeria. Fitoterapia.

[92] Yu H.S., Lee S.Y., Jang C.G. (2007). Involvement of 5-HT1A and GABAA receptors in the anxiolytic-like effects of *Cinnamomum cassia* in mice. Pharmacol. Biochem. Behav..

[93] Ravizza R., Gariboldi M.B., Molteni R., Monti E. (2008). Linalool, a plant-derived monoterpene alcohol, reverses doxorubicin resistance in human breast adenocarcinoma cells. Oncol. Rep..

[94] Kilani S., Abdelwahed A., Ben Ammar R. (2008). Chemical Composition of the Essential Oil of *Juniperus phoenicea* L. from Algeria. J. Essent. Oil..

[95] Moon T., Wilkinson J.M., Cavanagh H.M. (2006). Antiparasitic activity of two *Lavandula* essential oils against *Giardia duodenalis*, *Trichomonas vaginalis* and *Hexamitainflata*. Parasitol. Res..

[96] Priestley C.M., Burgess I.F., Williamson F.M. (2006). Lethality of essential oil constituents towards the human louse, *Pediculus humanus*, and its eggs. Fitoterapia.

[97] Rim I.S., Jee C.H. (2006). Acaricidal effects of herb essential oils against *Dermatophagoides farinae* and *D. pteronyssinus* (Acari: Pyroglyphidae) and qualitative analysis of a herb *Mentha pulegium* (pennyroyal). Korean J. Parasitol..

[98] Macías F.A., Chinchilla N., Varela R.M., Molinillo J.M. (2006). Bioactive steroids from *Oryza sativa* L.. Steroids.

[99] Singh H.P., Kaur S., Mittal S., Batish D.R., Kohli R.K. (2009). Essential oil of *Artemisia scoparia* inhibits plant growth by generating reactive oxygen species and causing oxidative damage. J. Chem. Ecol..

[100] Tellez M.R., Kobaisy M., Duke S.O., Schrader K.K., Dayan F.E., Romagni J., Kuo T.M., Gardner H.W. (2002). Terpenoid based defense in plants and other organisms. Lipid Technology.

[101] Angelini L.G., Carpanese G., Cioni P.L., Morelli I., Macchia M., Flamini G. (2003). Essential oils from Mediterranean Lamiaceae as weed germination inhibitors. J. Agric. Food Chem..

[102] Santos S., Moraes M.L.L., Rezende M.O.O., Souza-Filho A.P.S. (2011). Potencial alelopático e identificação de compostos secundários em extratos de calopogônio (*Calopogonium mucunoides*) utilizando eletroforese capilar. Eclética Quím..

[103] Dudai N., Poljakoff-Mayber A., Mayer A.M., Putievsky E., Lerne H.R. (1999). Essential oils as allelochemicals and their potential use as bioherbicides. J. Chem. Ecol..

[104] De Oliveira C.M., das Graças Cardoso M., Ionta M., Gomes Soares M., de Andrade Santiago J., Ferreira Da Silva G.A., Teixeira M.L., de Carvalho Selvati Rezende D.A., Vieira de Souza R., Isac Soares L. (2015). Chemical Characterization and in vitro Antitumor Activity of the Essential Oils from the Leaves and Flowers of *Callistemon viminalis*. Am. J. Plant Sci..

[105] Saad L.M.M.G., Abdelgaleil S.A.M. (2014). Allelopathic Potential of Essential Oils Isolated from Aromatic Plants on *Silybum marianum*. Glob. Adv. Res. J. Agric. Sci..

[106] Astani A., Reichling J., Schnitzler P. (2010). Comparative study on the antiviral activity of selected monoterpenes derived from essential oils. Phytother. Res..

[107] Kotan R., Kordali S., Cakir A., Kesdek M., Kaya Y., Kilic H. (2008). Antimicrobial and insecticidal activities of essential oil isolated from Turkish *Salvia hydrangea* DC. Ex. Benth. Biochem. Syst. Ecol..

[108] De Almeida R., Fernando L., Fernando F., Mancini E., de Feo V. (2010). Phytotoxic Activities of Mediterranean Essential Oils. Molecules.

[109] Vokou D., Douvli P., Blionis G.J., Halley J.M. (2003). Effects of monoterpenoids, acting alone or in pairs, on seed germination and subsequent seedling growth. J. Chem. Ecol..

[110] Bouajaj S., Romane A., Benyamna A., Amri I., Hanana M., Hamrouni L., Romdhane M. (2014). Essential oil composition, phytotoxic and antifungal activities of *Ruta chalepensis* L. leaves from High Atlas Mountains (Morocco). Natl. Prod. Res..

[111] Tabana A., Saharkhiza M.J., Hadian J. (2013). Allelopathic potential of essential oils from four *Satureja* spp.. Biol. Agric. Hortic..

[112] Paluch G.E., Zhu J., Bartholomay L., Coats R.J. (2011). Amyris and Siam-wood Essential Oils: Insect Activity of Sesquiterpenes. Pestic. Househ. Struct. Resid. Pest Manag..

[113] Ahmed S.M., Eapen M. (1986). Vapour toxicity and repellency of some essential oils to insect pests. Indian Perfum..

[114] Mateeva A., Karov S. (1983). Studies on the insecticidal effect of some essential oils. Naushni Tr. Vissha Selskostop. Inst. Vasil Kolar. Plodiv..

[115] Hamraoui A., Regnault-Roger C. (1995). Oviposition and larval growth of *Acanthoscelides obtectus* Say (Col., Bruchidae) in regard to host and non-host plants from leguminosae family. J. Appl. Entomol..

[116] Regnault-Roger C., Hamraoui A. (1995). Fumigant toxic activity and reproductive inhibition induced by Monoterpenes upon *Acanthoscelides obtectus* Say (Coleoptera), bruchid of kidney bean (*Phaseolus vulgaris*). J. Stored Prod. Res..

[117] Maia M.F., Moore S.J. (2011). Plant-based insect repellents: A review of their efficacy, development and testing. Malar. J..

